# Continuous Glucose Monitoring–Enhanced eConsult Improves Clinical Outcomes in Adults Living With Diabetes in a Safety Net Primary Care Setting

**DOI:** 10.1155/jdr/5547910

**Published:** 2025-08-05

**Authors:** Samantha M. Siskind, Ryanne Dymek, Kathryn L. Fantasia, Katelyn O'Brien, Devin W. Steenkamp

**Affiliations:** ^1^Section of Endocrinology, Diabetes, Nutrition & Weight Management, Boston University Chobanian & Avedisian School of Medicine, Boston, Massachusetts, USA; ^2^Section of General Internal Medicine, Boston University Chobanian & Avedisian School of Medicine, Boston, Massachusetts, USA; ^3^Department of Pharmacy, Section of General Internal Medicine, Boston Medical Center, Boston, Massachusetts, USA

**Keywords:** continuous glucose monitoring, electronic consultation

## Abstract

**Background:** There is a shortage of endocrinologists providing diabetes care. Electronic consultation (eConsult) improves access to subspecialty care, but the evaluation of CGM-enhanced eConsults in routine clinical practice has not been reported. We evaluated clinical outcomes after implementing a CGM-enhanced eConsult program in a safety-net hospital primary care clinic.

**Methods:** We completed a retrospective observational study assessing the clinical impact of the eConsult program. Participants included 67 adults (≥ 18 years) living with diabetes, receiving primary care at Boston Medical Center (mean age 65 years, 40.3% male, 79.1% Black, and 92.5% Type 2 diabetes). Demographic, clinical, and CGM data were analyzed from the medical record and Abbott's LibreView and Dexcom's Clarity web-based applications. Descriptive outcomes within 6 months post-eConsult included time to eConsult completion, hemoglobin A1c (HbA1c) change, medication adjustments, CGM prescription rates, and CGM-derived hypoglycemic metrics.

**Results:** Mean time to eConsult completion was 5.8 days. Endocrinologist recommendations were implemented in 86.6% of patients at the first primary care visit post-eConsult and in 94.0% of patients within 6 months. Within 6 months, HbA1c was unchanged (mean change 0.2% ± 0.4%). Relative to baseline, sulfonylurea prescription decreased 55.6%. The percentage of those prescribed basal insulin was unchanged, but basal insulin doses decreased in 41.8% of patients. Bolus insulin prescription increased 56.3% relative to baseline. Absolute CGM prescriptions increased from 2.9% at baseline to 49.3%. In 11 CGM users with sufficient CGM data for interpretation at 6 months, Level 1 hypoglycemia (54–69 mg/dL) decreased by 2% and Level 2 hypoglycemia (< 54 mg/dL) decreased by 0.7%.

**Conclusion:** In adults living with diabetes cared for in a safety-net hospital, CGM-enhanced eConsult provides timely access to endocrinologist expertise, recommendations are widely implemented by primary care clinicians, and guideline-directed modern diabetes therapeutic use increases, including a 17-fold increase in personal CGM prescriptions.

## 1. Introduction

Diabetes mellitus (DM) is an increasingly prevalent chronic condition that carries a significant burden for patients and disproportionately impacts patients of ethnic and racial minority backgrounds [1, 2]. Despite the increasing prevalence of DM, the United States faces a shortage of endocrinologists, who are specifically trained to provide specialty diabetes care. This shortage is particularly acute in rural and underserved parts of the United States [3]. Additionally, patients of minority backgrounds are referred to specialty care at lower rates than white patients [4]. Limited access to diabetes specialty care may further amplify the disparities in disease burden between white and nonwhite patients. Strategies that may positively impact diabetes care delivery in populations with reduced access to specialty care include the development of the primary care workforce, Project ECHO-like interventions [5], and provider-to-provider electronic consultations (eConsults). eConsults are a method of asynchronous communication between a primary care clinician and a specialist that allows the primary care clinician to seek specialist advice without the need for an in-person consultation. eConsults have been reported to be acceptable to both patients and clinicians, improve access to subspecialty care, and reduce the need for in-person consultation [6–8]. Diabetes technology, including office-applied professional continuous glucose monitoring (CGM), which allows for the collection of glucose data in a patient-blinded fashion for later review by a clinician, may enhance the ability to provide diabetes care recommendations for patients via eConsult, but there is limited research assessing the effectiveness of this type of referral method.

In 2018, to improve access to endocrinology expertise for patients with diabetes at our safety-net institution, a 6-month clinical pilot program introduced the use of professional CGM-enhanced endocrinology eConsults as a new referral option for patients receiving longitudinal diabetes care within the primary care setting. Data from the pilot program was previously published in 2021 and showed that CGM-enhanced eConsults resulted in more timely access to endocrinology expertise, were acceptable to patients, and resulted in similar short-term glycemic outcomes compared to in-person consultation [9]. However, the number of eConsults performed during the pilot study period was small. Since then, we have continued to provide CGM-enhanced eConsults as part of routine clinical care, and a larger number of patients have now been reached by this innovative referral method. In this follow-up study, we sought to further assess the impact of CGM-enhanced eConsults on patients receiving diabetes care in the primary care setting. We hypothesized that the eConsults would result in timely access to specialist recommendations, implementation of specialist recommendations by primary care clinicians, an increase in prescription of guideline-recommended therapy (e.g., SGLT2 inhibitors and GLP-1 receptor agonists), and improvement in glycemic control as measured by hemoglobin A1c (HbA1c) and CGM data, when available.

## 2. Materials and Methods

### 2.1. Setting

Boston Medical Center (BMC) is the largest academic safety-net hospital in New England. Over 60% of BMC patients are from minoritized groups, including racial and ethnic minority groups and public health insurance beneficiaries. In-person referral for endocrinology consultation is available to primary care clinicians for specialty diabetes care. However, in-person referral is often complicated by long wait times and a missed appointment rate of nearly 50% for new diabetes referrals.

### 2.2. eConsult Process

In the BMC General Internal Medicine (GIM) primary care clinic, a CGM-enhanced endocrinology eConsult was implemented as a referral option for primary care clinicians and clinical pharmacists, who collaborate on diabetes care in the GIM clinic. For patients referred for CGM-enhanced eConsult, a professional CGM (Freestyle Libre Pro, Abbott Diabetes Care, Alameda, CA) is placed by a clinical pharmacist. The Freestyle Libre Pro is a factory-calibrated interstitial glucose sensor that measures interstitial fluid glucose levels every 15 min and stores this data for up to 14 days. These sensors do not provide real-time data for patients to view but are later downloaded for retrospective review. The eConsult process has been described previously [9]. CGM analysis and eConsult recommendations were provided by one of three endocrinologists. Individualized eConsult recommendations were made using information provided in the consult order, medication list, and electronic medical record (EMR) data.

### 2.3. Study Design

This retrospective cohort study describes the impact of the CGM-enhanced endocrinology eConsult referral program on changes to medication and personal CGM prescription, timeliness of eConsults, and changes in glycemia. The primary outcome was the percentage of patients for whom a recommended clinical change in diabetes management was made from the eConsult. This was defined as a prescription change (e.g., new medication start, medication discontinuation, medication dose change, or prescription for personal CGM) implemented by the primary care clinician that corresponded with a recommendation made in the eConsult. Secondary descriptive outcomes within 6 months after eConsult included time between eConsult order and eConsult completion, change in HbA1c between baseline and 6 months, medication adjustments, CGM prescription rates, and CGM-derived hypoglycemic metrics in those individuals actively using personal CGM at 6 months. Adult (age ≥ 18 years) patients who had a CGM-enhanced endocrinology eConsult ordered by a primary care clinician or clinical pharmacist for diabetes management recommendations between the dates of April 1, 2023, and January 23, 2024, were included. This study was reviewed and approved by the Boston University Institutional Review Board.

### 2.4. Data Collection

Data were collected at baseline (up to 6 months before CGM-enhanced eConsult was ordered), on the date of the first primary care clinic visit after eConsult was completed, and 6 months after the eConsult was ordered. Data including demographics, body mass index (BMI), HbA1c, antihyperglycemic medications, personal CGM prescription, DM-related complications and comorbidities, dates that eConsults were ordered and completed, and dates of follow-up primary care clinic appointments were collected from the EMR. DM-related complications and comorbidities including retinopathy, neuropathy, atherosclerotic cardiovascular disease (ASCVD), and heart failure were identified by ICD-10 codes on problem lists and/or written documentation in clinician notes; renal complications were similarly identified and included labs demonstrating proteinuria or glomerular filtration rate (GFR) < 60 mL/min/1.73 m^2^. Additionally, we reviewed personal CGM data in the primary care clinic LibreView and Dexcom Clarity population database accounts and collected data on CGM use, average glucose level, glucose management indicator (GMI), glucose variability (GV), time in range (TIR), time above range (TAR), and time below range (TBR) at the time of eConsult and within the 6 months after eConsult was ordered in patients using personal CGM.

### 2.5. Data Analysis

Quantitative data are described using a simple descriptive analysis. Continuous data are reported as median and interquartile range (IQR) and categorical data as relative percentages. Formal statistical comparison testing was not performed given the small sample size in most subcategories.

## 3. Results

### 3.1. Participant Characteristics

During the study period, 72 CGM-enhanced eConsults were ordered for 70 individual patients. Two patients had two separate eConsults placed at different times during the study period; data was collected only for their initial eConsults. Three of the 70 patients had eConsults ordered but were subsequently lost to follow-up and were not seen in the primary care clinic after the eConsult was completed, and so they were excluded from the study. The primary indications for eConsult included concern for unrecognized hypoglycemia, discordant HbA1c and self-monitoring blood glucose data, and hyperglycemia. Baseline characteristics of the patients who received eConsults are shown in Table 1.

### 3.2. Clinical Outcomes

At least one recommended clinical change in diabetes management was made in 58/67 (86.6%) of patients at the first primary care clinician visit post-eConsult and in 63/67 (94.0%) of patients 6 months after the eConsult was ordered. Specific per-patient changes in prescriptions are shown in Table 2. A total of 144 prescription-related recommendations were made in the 67 eConsults, with an average of 2.1 (1.1–3.2) prescription-related recommendations made per eConsult. Of the 144 recommendations made, 104 (72.2%) were implemented by the end of the study period. Specific data regarding this per-recommendation data are shown in Table S1. A recommendation could include a new drug start, drug discontinuation, dose change, or replacing one medication with another. In the case of a recommendation to replace one medication with another, this was counted as a single recommendation for the total recommendation count; however, the recommendation for drug start and the recommendation for drug discontinuation were tallied separately, which explains the discrepancy in the total number of recommendations described in Table 3. A total of 47 behavior-related recommendations (e.g., recommendations regarding dietary modifications and timing of when to take medications) were made in the 67 eConsults; due to limitations in clinician documentation, we were unable to discern how many of these behavior-related recommendations were implemented. Glycemic control, as measured by HbA1c, was not different 6 months after the eConsult was ordered: 8.6% (7.0–10.2) at baseline versus 8.8% (6.8–10.8) at 6 months. The average time between when the eConsult was ordered and when it was completed was 5.8 days. There was an average of 41 days between when the eConsult was ordered and when the patient was seen again in the primary care clinic for diabetes management. Six patients (9%) were referred to be seen in person in the endocrinology clinic after the completion of the eConsult.

Six months after eConsult was ordered, relative to baseline, sulfonylurea prescription decreased by 55.6%. SGLT2 inhibitor prescription increased by 39.1%, and GLP-1 receptor agonist prescription was unchanged. No patients were prescribed a dual GLP-1/GIP receptor agonist at baseline; three patients (4.5%) were prescribed a dual GLP-1/GIP receptor agonist 6 months after consult. Twelve patients (17.9%) were newly started on one of these guideline-directed therapies (either an SGLT2 inhibitor, GLP-1 receptor agonist, or dual GLP-1/GIP receptor agonist) by the end of the 6-month period as compared to baseline. Additionally, some patients who were newly prescribed GLP-1 receptor agonists after eConsult recommendations subsequently discontinued these agents before the end of the study period. For patients not prescribed GLP-1 receptor agonists at 6-month follow-up, despite eConsult recommendations, the reasons for not prescribing or for discontinuing the medication included side effects, low BMI, history of pancreatitis, patient preference to avoid injection, allergy, and cost limitations. All SGLT2 inhibitors and GLP-1 receptor agonists started during the study period were prescribed by clinicians in the primary care clinic, with chart documentation indicating that these were being prescribed for improved glycemic control and/or renoprotection.

The frequency of basal insulin prescription was similar at baseline and 6 months after eConsult was ordered. The relative increase in bolus insulin prescription was 56.3% at the 6-month follow-up. In some cases, insulin doses were started and then discontinued during the 6-month study period; if a dose was started at any point during the study period, it was counted as a dose start at the 6-month mark. Patterns of insulin prescription are described in Table 3. Notably, basal insulin doses were decreased in 26 (38.8%) patients by the end of the first visit post-eConsult and in 28 (41.8%) patients 6 months after eConsult. At least one newly added dose of short-acting or “bolus” insulin was prescribed in eight (11.9%) patients by the end of the first visit post-eConsult and in 13 (19.4%) patients 6 months after eConsult.

At baseline, only two (2.9%) patients were prescribed a personal CGM. After the first primary care visit post-eConsult, 22 (32.8%) patients had been prescribed a personal CGM. Six months after eConsult, 33 (49.3%) patients were prescribed a personal CGM—a 17-fold increase from baseline. Six months after eConsult, 66.7% (22/33) of these patients shared their personal CGM data with their clinicians (available to review in Abbott's LibreView or Dexcom's Clarity web-based software). However, less than half (11/23, 47.8%) had sufficient data available to compare CGM metrics between baseline and at study end (defined as ≥ 70% sensor activity at their last available 14-day ambulatory glucose profile.)

Of this group with data sharing enabled and sufficient data for review, 81.8% (9/11) spent at least 15 min/day (> 1%) in the Level 1 hypoglycemic range (54–69 mg/dL) on the baseline CGM analysis. Six months after eConsult, only 27.3% (3/11) spent > 1% in Level 1 hypoglycemia. The average absolute decrease in Level 1 hypoglycemia was 2% and the average absolute decrease in Level 2 hypoglycemia (< 54 mg/dL) was 0.7%. Only one patient increased TBR (< 70 mg/dL).

## 4. Discussion

This retrospective cohort study describes the clinical impact of a CGM-enhanced endocrinology eConsult program implemented within a primary care clinic in a large academic safety-net hospital system. eConsults were an efficient method of obtaining endocrine specialty expertise, and recommendations were widely implemented, with at least one recommended clinical change being implemented in 94% of patients who received eConsults by the end of the study period. Additionally, eConsults increased the use of evidence-based therapies with cardiorenal benefits and reduced the use or doses of medications with increased risk of hypoglycemia. Notably, nearly 20% of patients who received an eConsult were started on an SGLT2i, GLP-1 receptor agonist, or dual GIP/GLP-1 receptor agonist, and there was a 17-fold increase in personal CGM use within 6 months.

Similar to previous descriptions of eConsult in endocrinology and diabetes care, the time between when the eConsult was ordered and when it was completed was significantly shorter than the average time between in-person referral and endocrinologist appointment [9–12]. Of the 67 patients who received eConsult recommendations, 92.9% identified as Black or Hispanic, with 79% identifying as Black or African American. This is a much higher percentage of racial and ethnic minority group patients than what is representative of our hospital system's demographics [9]. It is unclear why a higher number of patients from these racial and ethnic groups were referred for eConsult but suggests that CGM-enhanced eConsults may be a valuable strategy for improving access to endocrinology expertise for patients of historically marginalized racial and ethnic groups. While previous data has demonstrated lower rates of specialty referrals (other than endocrinology) for Black as compared to white people [4], it is unclear if racial inequities in the use of eConsult exist. It is plausible that eConsults may help to address (or are perceived by clinicians to help to address) some of the socioeconomic barriers that disproportionately impact people from minoritized backgrounds and that negatively impact in-person specialty consultation, including travel costs, additional copayments for clinic visits, and time off from work, though this should be further evaluated.

Additionally, the average age of the patients who received eConsult recommendations was 65 (53.8–76.1) years. This may represent an older demographic of patients compared to the more varied age range of patients who are often seen for in-person consultations. eConsult may be a valuable option for older adults who may have more difficulty getting to in-person specialty appointments due to challenges with mobility or transportation. Additionally, it is possible that primary care clinicians sought specialist advice for older patients at a higher frequency than younger patients, as older patients often have more comorbidities that may add complexity to their care. There is existing literature that does demonstrate that eConsult services provide benefit to older patients by reducing wait times and improving access to care [13].

An average of 2.1 prescription changes was recommended per eConsult, and 72.2% of changes were implemented by 6 months after eConsult was ordered. At least one recommended change was made in the diabetes management plan for 86.6% of patients after the first visit post-eConsult and in 94% of patients 6 months after eConsult. The majority of the recommended changes were to start a new medication or adjust a medication dose. Notably, while the frequency of patients prescribed GLP-1 receptor agonist therapy did not change during the study period, five (7.5%) patients were newly started on GLP-1 receptor agonists, and the dose of an already prescribed GLP-1 receptor agonist was increased in nine (13.4%) patients in accordance with eConsult recommendations. The apparent lack of change in patients prescribed this class of medication at baseline and by the end of the study period may be confounded by the fact that some individuals who were newly prescribed GLP-1 receptor agonists after eConsult subsequently discontinued before the end of the study period. Low rates of use of dual GLP-1/GIP receptor agonists both before and after eConsult are likely a result of this medication option only recently becoming covered by Massachusetts Medicaid (the insurance coverage for a majority of patients seen in our institution) shortly before the start of the study period. However, of the three dual GLP-1/GIP receptor agonists that were prescribed, two were prescribed in accordance with eConsult recommendations. Interestingly, there was a high relative increase in SGLT2 inhibitor prescription during the study period, despite SGLT2 inhibitors being formally recommended in the eConsult in only three of the patients for whom it was newly started. Over 25% of patients were prescribed sulfonylurea therapy at baseline, which was reduced by over 50% at 6 months. This guideline and evidence-based recommendation aligns with recent recommendations to de-emphasize nonincretin secretagogue therapies in favor of incretin secretagogues and SGLT2 inhibitors [14]. The frequency of basal insulin prescription was similar at baseline and post-eConsult. However, basal insulin doses decreased, while prescription of bolus insulin doses increased. This suggests that the CGM data may have helped identify nocturnal hypoglycemia or other glycemic metrics suggestive of excessive basal insulin dosing [15], where patients may benefit from reducing their basal insulin dose and possibly from adding bolus insulin doses or other antihyperglycemic therapies to address unrecognized postprandial hyperglycemia. Given the reduction in basal insulin doses without change in HbA1c, we hypothesize that insulin dose reduction and sulfonylurea discontinuation may have been recommended in response to previously unrecognized CGM-detected hypoglycemia.

An important clinical change arising out of this program was the dramatic increase in the prescription of personal CGM. In some cases, personal CGM was prescribed in accordance with endocrinologist eConsult recommendations. However, in other cases, it was prescribed by the clinical pharmacist upon removal of the professional CGM sensors, before endocrinologist recommendations had been received. This suggests that patient and/or primary care clinician exposure to CGM may have increased comfort and revealed previously unrecognized value to the use of CGM, thereby increasing the uptake of personal CGM use without explicit recommendations to begin on CGM from an endocrinologist. It is important to acknowledge that Medicaid insurance coverage for personal CGM is excellent in Massachusetts, which likely facilitated the uptake of CGM in this cohort [16].

Compared to prior studies of eConsult in diabetes care, our study further supports the understanding that eConsults reduce the time to specialty consultations, reduce the patient costs of travel, and facilitate timely therapeutic change [12]. Most patients had at least one prescription adjustment at follow-up and were consistent with our prior small pilot report on this topic [9]. We again report that professional CGM-enhanced eConsults may allow de-escalation of therapies carrying risk for hypoglycemia. In contrast to our previous work and that of others [9, 12], we did not observe an improvement in HbA1c 6 months after eConsult completion.

This study does have limitations. While this study reports outcomes from a larger cohort of CGM-enhanced eConsults conducted during routine primary care, given the observational nature of this study, data should be interpreted with caution. Patients who received CGM-enhanced eConsult likely differed systematically from those who were referred to endocrinology in person and did differ from those who were managed by primary care alone. While the CGM-enhanced eConsult was designed and implemented as an attempt to overcome traditional barriers to specialty diabetes consultation for a safety-net setting that cares for a large population who have a disproportionate burden of adverse social determinants of health [17], given the lack of a control group, we cannot determine the effectiveness of CGM-enhanced eConsults as compared to usual care, both with respect to glycemia and receipt of specialty diabetes recommendations. While professional CGM was used to make initial changes in therapies, repeat professional CGM was not completed and only a relatively small number of patients were using personal CGM with sufficient sensor data available to review at 6 months to be able to assess individual glycemic changes 6 months after eConsult. The reasons for limited sensor data available for review is unclear, but patients and primary care teams may benefit from further education and support on CGM use. Finally, data was collected for up to 6 months after eConsult was completed, and we cannot draw longer term conclusions with regard to most of our outcomes.

## 5. Conclusion

In an adult population living with diabetes and cared for in a safety-net hospital, CGM-enhanced eConsult provides timely access to endocrinologist expertise and recommendations that are widely implemented by primary care clinicians. Additionally, in patients who received eConsults, the prescription of guideline-directed modern diabetes therapeutics increased, medications associated with hypoglycemia risk were discontinued or doses were reduced, and personal CGM prescription increased. Future studies should assess the clinical effectiveness and cost effectiveness of CGM-enhanced eConsults.

## Figures and Tables

**Table 1:** Baseline characteristics of participants.

**n**	**67**
Age (years)	65.0 (53.8–76.1)
Sex, male	27 (40.3%)
Initial HbA1c (%)	8.6 (7.0–10.2)
Race/ethnicity	
Black	53 (79.1%)
White	1 (1.5%)
Asian	2 (3.0%)
Hispanic, Latino	9 (13.4%)
Other	2 (3.0%)
BMI (kg/m^2^)	29.2 (23.3–35.1)
Obese (BMI ≥ 30 kg/m^2^)	25 (37.3%)
Type 2 diabetes mellitus	62 (92.5%)
Steroid-induced hyperglycemia	3 (4.5%)
Type 1 diabetes mellitus	2 (3.0%)
Renal complication	39 (58.2%)
Retinopathy	19 (28.4%)
Neuropathy	44 (65.7%)
ASCVD	14 (20.9%)
Heart failure with reduced ejection fraction	4 (6.0%)
Heart failure with preserved ejection fraction	3 (4.5%)

*Note: N* (percent) for categorical data and median (IQR) for the continuous data. Renal complication is defined as the presence of proteinuria or chronic kidney disease (by chart diagnosis or eGFR < 60 mL/min/1.73m^2^).

Abbreviation:ASCVD, atherosclerotic cardiovascular disease.

**Table 2:** Frequency of noninsulin prescriptions.

	**Baseline**	**First visit postconsult**	**6 months postconsult**
**N**	**67**	**67**	**67**
Metformin	41 (61.2%)	40 (59.7%)	38 (56.7%)
Started as per recommendation			0
Stopped as per recommendation			1
Dose change as per recommendation			1 dose decrease
Sulfonylurea	18 (25.7%)	11 (16.4%)	8 (11.9%)
Started as per recommendation			1
Stopped as per recommendation			9
Dose change as per recommendation			1 dose decrease
Meglitinide	1 (1.4%)	1 (1.5%)	1 (1.5%)
Started as per recommendation			1
Stopped as per recommendation			0
Dose change as per recommendation			0
Thiazolidinedione	1 (1.4%)	1 (1.5%)	1 (1.5%)
Started as per recommendation			0
Stopped as per recommendation			0
Dose change as per recommendation			0
DPP4 inhibitor	9 (12.9%)	10 (14.9%)	10 (14.9%)
Started as per recommendation			4
Stopped as per recommendation			2
Dose change as per recommendation			0
GLP-1 receptor agonist	30 (44.8%)	36 (53.7%)	31 (46.3%)
Started as per recommendation			5
Stopped as per recommendation			1
Dose change as per recommendation			9 dose increases
Dual GLP-1/GIP receptor agonist	0 (0%)	0 (0%)	3 (4.5%)
Started as per recommendation			2
Stopped as per recommendation			0
Dose change as per recommendation			0
SGLT2 inhibitor	23 (34.3%)	28 (41.8%)	32 (47.8%)
Started as per recommendation			3
Stopped as per recommendation			1
Dose change as per recommendation			0
Personal CGM	2 (2.9%)	22 (32.8%)	33 (49.3%)
Started as per recommendation			10
New GLP-1 receptor agonist, GLP-1/GIP receptor agonist, and/or SGLT2 inhibitor started	N/A	10 (14.9%)	12 (17.9%)
Started as per recommendation			10

*Note:* Data are presented as *n* (percent). Percentages include the entire cohort of 67 patients who attended the first visit post-eConsult.

**Table 3:** Frequency of insulin prescription and dose titration.

	**Baseline**	**First visit postconsult**	**6 months postconsult**
Basal insulin	46 (68.7%)	48 (71.6%)	46 (68.7%)
Bolus insulin	16 (23.9%)	21 (31.3%)	25 (37.3%)
New basal dose started		5 (7.5%)	4 (6.0%)
Started as per recommendation			5
New bolus dose started		8 (11.9%)	13 (19.4%)
Started as per recommendation			11
Basal dose increased		4 (6.0%)	5 (7.5%)
Increased as per recommendation			2
Basal dose decreased		26 (38.8%)	28 (41.8%)
Decreased as per recommendation			25
Bolus dose increased		3 (4.5%)	4 (6.0%)
Increased as per recommendation			2
Bolus dose decreased		3 (4.5%)	4 (6.0%)
Decreased as per recommendation			5
Basal insulin discontinued		3 (4.5%)	4 (6.0%)
Discontinued as per recommendation			2
Bolus insulin discontinued		1 (1.5%)	1 (1.5%)
Discontinued as per recommendation			2

*Note:* Data are presented as *n* (percent). Percentages include the entire cohort of 67 patients who attended the first visit post-eConsult.

## Data Availability

The data that support the findings of this study are available from the corresponding author upon reasonable request.
