# Supplementary material for: Continuous Glucose Monitoring–Enhanced eConsult Improves Clinical Outcomes in Adults Living With Diabetes in a Safety Net Primary Care Setting
Source: J Diabetes Res. 2025 Aug 5;2025:5547910. doi: 10.1155/jdr/5547910 (PMC12343161; doi:10.1155/jdr/5547910)
Supplement: Supporting Information — Additional supporting information can be found online in the Supporting Information section. [file 5547910.f1.docx]

**Supplement**

TABLE 1

|  | Number of Changes Recommended in eConsult | Number of Changes Implemented at 6 Months |
| --- | --- | --- |
| Total | 144 | 104 (72.2%) |
| Continue medication dose | 11 | 8 (72.7%) |
| Start new medication | 61 | 38 (62.3%) |
| Discontinue medication | 28 | 19 (67.9%) |
| Change medication dose | 47 | 41 (87.2%) |
| Dose increase | 33 | 29 (87.9%) |
| Dose decrease | 14 | 12 (85.7%) |
| Prescribe personal CGM | 14 | 10 (71.4%) |

Data presented as n (%)
